# Are Zirconia Bioceramics and Ceramics Intended to Come in Contact with Skin Inert?

**DOI:** 10.3390/ma13071697

**Published:** 2020-04-05

**Authors:** Lucien Reclaru, Lavinia Cosmina Ardelean, Catalin Adrian Miu, Alexandru Florian Grecu

**Affiliations:** 1 Scientific Consultant Biomaterials and Medical Devices, 103 Paul-Vouga, 2074 Marin-Neuchâtel, Switzerland; lreclaru@gmail.com; 2 Department of Technology of Materials and Devices in Dental Medicine, “Victor Babes” University of Medicine and Pharmacy Timisoara, 2 Eftimie Murgu sq, 300041 Timisoara, Romania; 3 Department of Orthopaedics-Traumatology, “Victor Babes” University of Medicine and Pharmacy Timisoara, 2 Eftimie Murgu sq, 300041 Timisoara, Romania; miu.catalin@umft.ro; 4 Department of Orthopaedics and Traumatology, University of Medicine and Pharmacy Craiova, 2 Petru Rares str, 200349 Craiova, Romania; alexandru.grecu@umfcv.ro

**Keywords:** bioceramics, zirconia ceramic, cation extraction, dental ceramic, jewelry and watchmaking ceramics, toxicity, ceramics dissolution, cations release from ceramics

## Abstract

Generally speaking, ceramic materials are insensitive to corrosion, compared to most other materials. The present study questions the fact that ceramics are inert. Two major aspects are to be considered: the stability of zirconia over time, the stable tetragonal phase transforming into an unstable monoclinic form; the multitude of manufacturing methods, using various additives, sintering additives, oxides mixing, impurities, grain boundaries, and porosities which strongly influence the corrosion behavior and chemical degradation. In case of the investigated ceramics two paths were pursued:a) Dissolutions of ceramics in a mixture of HNO_3_ 60% and HF 40% ultrapure medium.b) Release of cations from ceramics in various mediums:dental bioceramics in a 0.07 M HCl medium and a 0.1% NaF+0.1% KF medium; ceramics used in jewelry and watchmaking applications in a HCl 0.07 M medium and an artificial sweat medium. By inductively coupled plasma-optical emission spectrometry/mass spectrometry (ICP-OES/MS), traces of significant chemical elements were assessed: Hf, Cr, Y, As, Pb, Al, Fe, Cu, Se, Sb, La, Ni, Co, Sb, Ta, Te, Ba, Sm, Nb, Hg, Cd, Sr, As and Se. In ceramics used in jewelry and watchmaking applications the concentrations found vary from one ceramic to another, including toxic elements such as Te, Ba, As, Pb, Sm, Hg and Cd, therefore being technical zirconia ceramics which are not intended for the medical field. For ceramics used in jewelry and watchmaking applications a screening identification test for Ni, Co, Cu and Fe with strips of type Merckoquant^®^ (Merck, Kenilworth, NJ, USA) was also performed. The obtained data prove that the zirconia ceramics in question are far from being “inert”.

## 1. Introduction

The definition of a ceramic material as given by ASTM C242-12 [1] is: “An article having a glazed or unglazed body of crystalline or partly crystalline structure, or of glass, which body is produced from essentially inorganic, nonmetallic substances and either is formed from a molten mass which solidifies on cooling or is formed and simultaneously or subsequently matured by the action of the heat.”

A more complex definition has been formulated by CTIOA: “A ceramic is an inorganic, nonmetallic solid prepared by the action of heat and subsequent cooling.” Ceramic materials may have a crystalline or partly crystalline structure or may be amorphous (glass). Because most common ceramics are crystalline, the definition of ceramics is often restricted to inorganic crystalline materials, as opposed to the noncrystalline glasses [2].

Ceramics are classified in three different categories:

- oxides: aluminum oxide, zirconium oxide;
- non-oxides: carbides, borides, nitrides, ceramics composed of silicate and atoms such as tungsten, magnesium, platinum, or even titanium;
- composite ceramics: combination of oxides and non-oxides.

### 1.1. Dental Ceramics

The term “bioceramics” may be applied to the category of biomaterials that are composed of ceramic as one of its constituents. These materials are characterized by biocompatibility with human tissue, and have wide applications in the medical and dental field. They have been used in repair and replacement of the musculoskeletal organs, as bone substitutes, bone implants and artificial joints [3,4].They are also used in making artificial heart valves [5]. In dental applications, zirconia, due to its mechanical properties and excellent biocompatibility, is considered as the material of choice for fixed restorations [6]. Zirconia frame works, slightly translucent, are considered more aesthetic because they are less visible than metallic Co–Cr ones. Other dental applications of bioceramics include implants, periodontal treatment and alveolar ridge augmentation. Zirconia is well tolerated by the oral soft tissues, resulting in long-term implant stability. The adhesion of fibroblasts and osteoblasts to zirconia has proven the integration with bone and soft tissue cells [5,7].

The types of ceramics used in medical applications are presented in Table 1.

**Figure 1:**
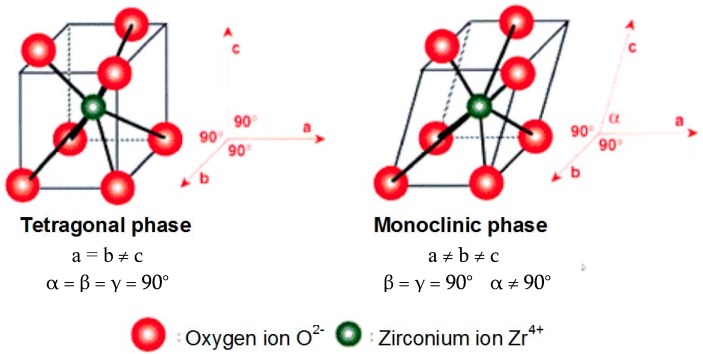
Tetragonal and monoclinic zirconium oxide (zirconia) structures (reconstructed by drawing with Lotus Smart Suite, Freelance Graphics IBM 2005, based on references [10,11]).

The zirconia crystals are organized in the form of a straight prism with a rectangular side, this being the tetragonal phase, and in the shape of a deformed prism with a parallelepiped side, as the monoclinic phase (Figure 1). The tetragonal or quadratic phase is stable, the monoclinic phase is unstable. Only the tetragonal phase allows obtaining a ceramic with satisfactory mechanical properties. The monoclinic phase weakens the mechanical performances and has a contribution in decreasing the cohesion of the ceramic grains and therefore of its density [8]. The tetragonal phase characterizes the pure zirconia at around 1000 °C, the monoclinic phase being dominant at room temperature [9]. To prevent tetragonal zirconia from turning into monoclinic phase at room temperature, approximately 5wt.%yttria (Y_2_O_3_) is added by weight as a stabilizing agent, therefore the name yttria stabilized tetragonal zirconia polycrystal (Y-TZP) [9]. Y-TZP is only metastable, allowing it to still have stress-induced phase transformation toughening, which gives zirconia its superior mechanical properties, but also allows tetragonal to monoclinic transformation over extended periods of time. This phase transformation is often referred to as low temperature degradation. At high temperatures (100 °C to 500 °C), in the presence of water or water vapors, the transformation process from Y-TZP to monoclinic phase is greatly accelerated, with a maximum transformation rate at 250 °C [9]. It is easily understandable why it is essential that the proportion of monoclinic phase has to be as low as possible. Consequently, the manufacturing and sterilization processes must limit the possibilities of passing from a stable tetragonal phase to an unstable monoclinic phase.

The samples taken as references are regularly treated in an autoclave for 5 h at 134 °C to simulate 20 years of aging in vivo. This results in the formation of only 2% to 3% of monoclinic phase, which is weak and apparently does not have any drawback in the in vivo functionality [12,13].

As for the composition of zirconia dental ceramic there are constraints which imply very important difficulties in their manufacture. The minimum purity of zirconia must be 93.6%. For good stability, the amount of yttrium oxide in ceramic should be 5.15%. The most common impurities are aluminum oxide or alumina, its level should be less than 0.5% (Table 2). The density of zirconia must be the closest to the theoretical density (100% dense), i.e., 6.1. The closer to this value, the less space between the grains, the greater mechanical resistance and lower roughness. The standards impose 6 [12,13,14,15], the ideal being 6.1. The ceramic grains must be smaller than 0.6 μm, as the resistance of the block and its surface condition depends on the grain size.

Large-scale results concerning the corrosion behavior of Y-TZP ceramics and its chemical degradation properties have not been reported yet.

### 1.2. CeramicsIntended to Come in Contact with Skin

In the world of jewelry, cubic zirconia acts as a substitute for diamonds and its applications include rings, bracelets, earrings, necklaces, etc., in other words, articles that are supposed to come in contact with the skin. Various technical ceramics are used in watchmaking, for producing watch cases, bracelets, etc. The material is valued because its light weight, scratch resistance, durability and smooth touch.

There is a large number of manufacturers of industrial ceramics on the market. Establishing the source of industrial ceramics is very difficult, their origin can be China, Japan, Korea or Europe. Manufacturers of ceramic articles are being opaque in disclosing information, for the reason of confidentiality and competition in specific markets. Various luxury watch brands use ceramics or black ceramics, as well as jewelry manufacturers.

At present Europe has a new vision regarding chemicals toxicology to humans, because of the REACH regulation (registration, evaluation, authorization, and restriction of chemicals) [16,17].

European Chemical Agency (ECHA) has developed a plan for the implementation of the substances of very high concern (SVHC) [18], namely endocrine disruptors (ED), carcinogens, mutagens, toxics for reproduction (CMR), and sensitizers (skin sensitizers and respiratory sensitizers).

Thus, the biocompatibility of materials in contact with a living tissue becomes a puzzle in the overall picture of evaluating the effects of chemicals toxicity which come into contact with humans [19,20,21].

Different chemicals from different sources, released at different moments and from different places, combine and expose humans to a mixture of chemicals, inducing the “cocktail effect”, which is a burning scientific subject [22,23], as individual chemicals may become more dangerous when mixed with other chemicals.

(ECHA) is trying to understand how the chemicals are released from different sources and how they combine with each other to give rise to a human exposure with adverse effects [24].

REACH Annexes VII [25] and VIII [26] contain the provision that acute studies do not need to be conducted if there are mitigating factors indicating that toxicity is unlike to occur, for instance if the substance is highly insoluble in water or the substance is unlikely to cross biological membranes. On the other hand, REACH has been asking manufacturers to consider long-term studies when the substance has low water solubility.

Under REACH concept if the substance appears “insoluble” in water, a limit test up to the detection limit of the analytical method shall be performed [27].

Ceramics corrosion behavior and chemical degradation are strongly influenced by the multitude of manufacturing methods, using various additives (such as sintering additives), oxides mixing, impurities, gas phases at grain boundaries, and porosities. The question is which products are being released from a ceramic and in which quantities. Today, mass spectroscopy enables analyzing quantities in ppb (µg/L) and to measure interfaces electric currents of 10^−15^ A magnitude.

In general, ceramic materials are insensitive to corrosion, compared to most other materials. In this context, the aim of this study is to assess whether ceramics are really inert. The research areas addressed are limited to dental applications and articles in contact with the skin.

In perspective, it is of great interest to understand the toxicokinetic phenomena of cations released as traces into the body. According to ECHA and Joint Research Centre (JRC), toxicokinetics relates to the penetration of a chemical into the body (for example oral, dermal or through inhalation) and its subsequent fate following exposure. Absorption is the process by which a compound passages from outer lining membranes such as the gut epithelium, the skin or the lung epithelium, subsequently reaching the fluids and tissues/organs of the body. Compounds can be absorbed passively through the cell membrane (transcellular route), via the junctions between the cells (paracellular route) or actively through carrier-mediation via an active or facilitated diffusion. The preferred pathway for absorption or transport of a specific compound depends on its physicochemical characteristics and the biological features of the membrane. The questions to be answered in the future concern which cations diffuse easily and which are the possible toxicological effects [28].

## 2. Materials and Methods

### 2.1. Zirconia for Dental Applications

**Figure 2:**
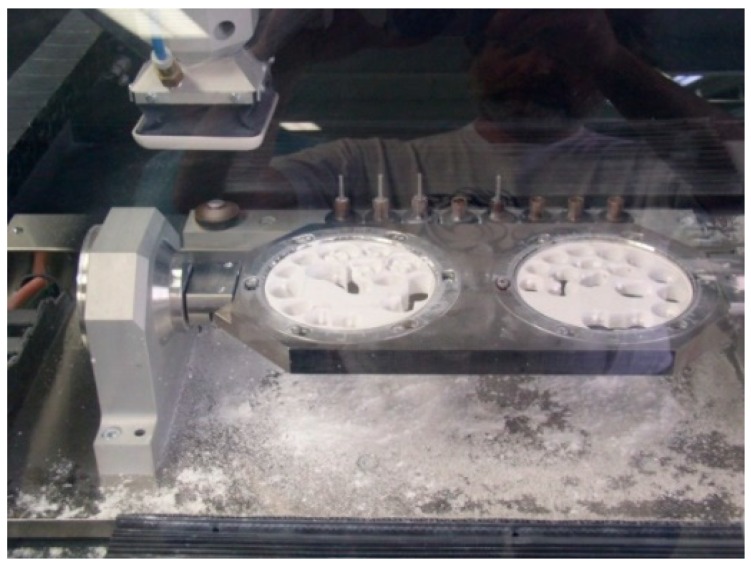
Milling machine Coritec250i (imes-icore).

**Figure 3:**
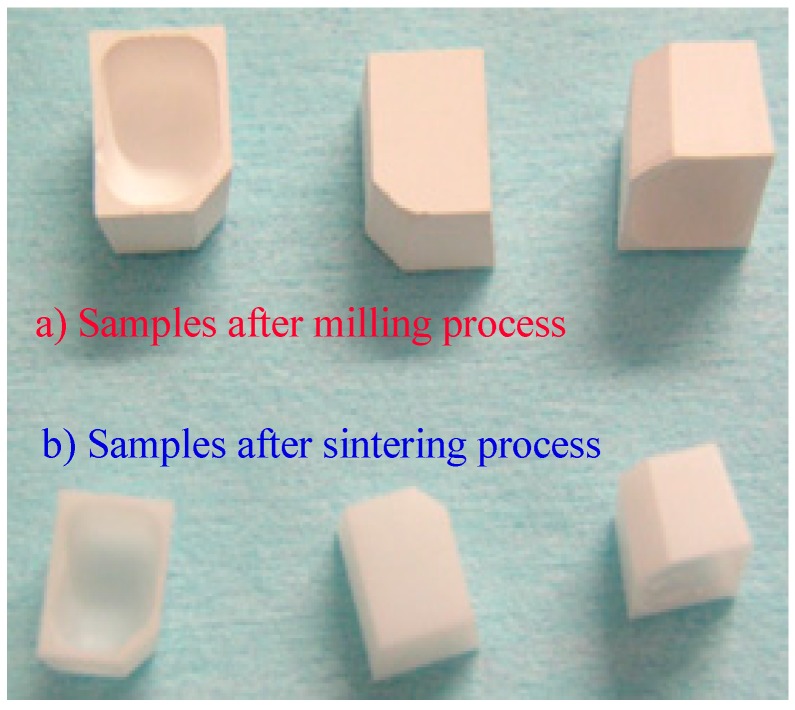
The samples.

The dental ceramic used for testing is produced by Metoxit (Thayngen, Switzerland). The samples were obtained from bulk by milling with a CAD/CAM Coritec 250i (imes-icore, Eiterfeld, Germany) 5 axes machine (Figure 2). The samples are presented in Figure 3a,b: (a) after milling and (b) after completing the sintering process at 1400 °C.

The release of cations was carried out in the 0.07 M HCl (ISO EN 71-3 2013) medium [26] and 0.1%NaF+0.1%KF. The solutions have been filtered before use over a sterilized Falcon^®^ 0.22 μm cellulose acetate membrane; the release flasks used being of Falcon^®^ sterile type made of polypropylene. The salts and water used are of ultrapure quality, according to the Merck classification.

The samples, of parallepipedicrectangular shape, have been firstly cleaned in ethanol p.a. under ultrasound, the ratio of release solution volume/total sample surface being equal to 1. The extraction has been carried out at 37 °C, shielded from light, for 168 h.

The 0.07 M HCl medium was chosen as it is commonly used for the evaluation of metals extracted from toys. Children, who are more susceptible to toxicological reaction problems than adults, often tend to suck the toys or even swallow the small component pieces. On the other hand, it is an excellent extraction medium with no particular problems of preparation or quantitative analysis, being commonly used for the analysis of the bioavailability of toxic elements such as As, Sb, Pb, Hg, etc. [29].

As for the 0.1%NaF+0.1%KF medium, its concentration is specific for the composition of toothpastes, so it has been of interest to assess the release of ceramic cations in this type of environment. This medium was used in the past for corrosion studies of titanium dental implants. The goal was to find out the effects of toothpaste in contact with the titanium dental implant [30].

For each environment test 3 samples have been used and 2 blank samples have been measured as references. The solutions have been analyzed by two cross techniques: inductively coupled plasma-optical emission spectrometry (ICP-OES) Optima 8300 (Perkin Elmer, Waltham, MA, USA) and inductively coupled plasma-mass spectrometry (ICP-MS)Elan DRC(Perkin Elmer, Waltham, MA, USA).

The cations matrices have been measured as follows:

ICP-OES: Al, Cr, Cu, Fe, Ni, P, S, Ti, V, Zn, As, Hg, Sb, Se.
ICP-MS: Ba, Cd, Co, Hg, Li, Mo, Nb, Pb, Sb, Sn, Sr, Zn, Hf, Y, Zr.

A statistical analysis of the released cations was performed for As, Ba, Cd, Co, Cr, Cu, Fe, Hf, Hg, Mo, Nb, Ni, Pb, Sb, Se, Te, Ti, V, W and Zr.

The maximum accepted risk for Type-I error (false rejection of the null hypothesis, leading to the erroneous conclusion that differences between ceramics exist) are:

Fe (±22%), Cr, Cu, Mo, Ni, and Co (±17%) in 0.07 M HCl.
Fe (±27%), Cr, Cu, Mo, Ni, and Co (±22%) in 0.1%NaF+0.1%KF.

### 2.2. Ceramics Intended to Come inContact with Skin

The tested materials are ceramic items that come from the jewelry sector (bracelets, necklaces, rings, earrings, etc.), watches and other kinds of jewelry. Their images are displayed in Section 3.2, for a better understanding of the results obtained.

Four types of ceramics used in contact with skin were assessed: a cermet, zirconia ceramics used in watchmaking applications, a black zirconia ceramic jewelry component and zirconia ceramic rings, jewelry components. 

As considered inert, ceramics evaluations in conformity with REACH toxicity, namely Very High Concern (SVHC), Endocrine Disrupters (ED), Carcinogens, Mutagens or Toxic to reproduction(CMR), and the sensitizers and allergens (SA), are often neglected. This is the reason why we choose to evaluate this matter, by trace analysis of the chemical composition of ceramics and cations release by extraction, in several categories of ceramics intended to come into direct or prolonged contact with skin. 

**Figure 4:**
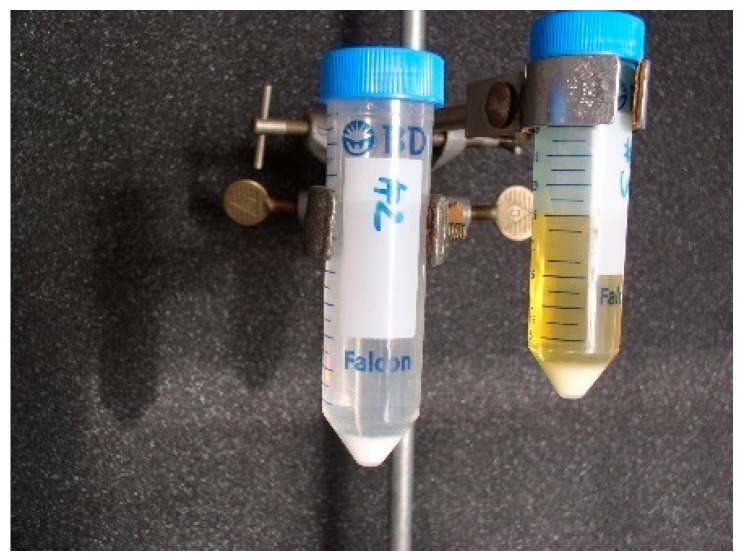
Dissolving ceramics in an acid medium HNO_3_+HF.

Their assessment is based on:

- dissolving ceramics in a mixture of HNO_3_ 60% and HF 40% ultrapure (Figure 4).

After filtration of the precipitates, the filtrates are diluted in 50 mL with Merck Ultrapur^®^ water, dried in the open air and analyzed by ICP. The scanning electron microscope (SEM) semi-quantitative analysis of powder (precipitate) from the filter paper was carried out.

The trace analysis has been performed by ICP-OES Optima 8300 (Perkin Elmer, Waltham, MA, USA) and ICP-MS Elan DRC (Perkin Elmer, Waltham, MA, USA).

Cations have been measured as follows: As, Ba, Cd, Co, Cr, Cu, Fe, Hf, Hg, Mo, Nb, Ni, Pb, Sb, Se, Te, Ti, V, W and Zr.

- extraction in appropriate media: HCl 0.07 M solution [31] and artificial sweat, followed by the analysis of the cations release by ICP-OES Optima 8300 (Perkin Elmer, Waltham, MA, USA) and ICP-MS Elan DRC (Perkin Elmer, Waltham, MA, USA).

Extraction in artificial sweat was done according to EN 1811-2011 [32] for 7 days at 30 °C. The composition of the extraction medium is: 1±0.01 g/L urea, 5±0.05 g/L NaCl, 1±0.01 g/L lactic acid, and pH = 6.5±0.05 via NaOH 0.1 M and deionized water 18 MΩ·cm.

The selection of the analyzed elements is based on the following considerations: they are likely to enter into the composition of the matrix (Fe, Co, Ni, Cu) or in carbides(Cr) or they have significant toxicity (As, Ba, Cd, Cr, Hg, Pb, Sb, Se, Te).

- screening identification test for Ni, Co, Cu and Fe with the strips type Merckoquant^®^ (Merck, Kenilworth, NJ, USA).

A fine powder of the ceramicto be analyzed has been removed by using an abrasive strip (grain 600), lightly rubbing it on the surface of the tested sample. The strip containing the fine powder is then introduced into a test tube containing 5 drops of a 4M hydrochloric acid solution. After 4h and 18h, a drop of 4M hydrochloric acid is taken in order to carry out the test on the strip type Merckoquant^®^ (Merck, Kenilworth, NJ, USA) corresponding to the investigated element. A drop of the 4 M hydrochloric acid extraction solution, taken from the test tube, is placed on the reactive part of the strip, previously soaked with a drop of 10% ammonia. The element’s concentration is determined semi-quantitatively by visual comparison of the reaction zone of the test strip with a colorimetric scale.

Ni (II) ions form a red complex with dimethylglyoxime. Measuring range: 10–500 mg/L.
Co (II) ions form with the thiocyanate ions a blue complex. Measuring range: 10–1000 mg/L.
Cu (II) ions are reduced by a mixture of reducers in copper (I) ions. These form a purple complex with 2,2′-biquinoline (cuproin). Measuring range: 10–300 mg/L.
Fe (II) ions together with 2,2′-bipyridine form a red complex. Measuring range: 3–500 mg/L.

## 3. Results and Discussion

### 3.1. Zirconia for DentalApplications

Two series of extractions were performed: (a) in 0.07 M HCl acid medium and (b) in fluoride medium KF0.1%+NaF0.1%. The codes 1, 2 and 3 represent the after CAD/CAM milling samples and codes 4, 5 and 6 the after-sintering samples. The results obtained are presented in Table 3. For better understanding, the values obtained were grouped in four graphs with concentration scales of µg/L.

ICP-OES: sampling solution 1 mL+9 mL ultrapure H_2_O.

ICP-MS: sampling solution 1 mL+100 mL calibration standard solution +8.9 mL ultrapure H_2_O.

Analyzed metals which are at the detection limit of the ICP: Ni<1 µg/L, Co<0.2 µg/L, Ta<0.2 µg/L, Te<1 µg/L, Ba<0.2 µg/L, Sm<1 µg/L, Nb<0.2 µg/L, Hg<0.5 µg/L, Cd<0.2 µg/L, and Sr<0.2 µg/L, have not been not included in Table 3.

The analysis of the overall results shows that the quantities of released cations depend on several factors: the physical state of the raw material, type of processing: milling or heat treatment (sintering) and the extraction medium used.

**Figure 5:**
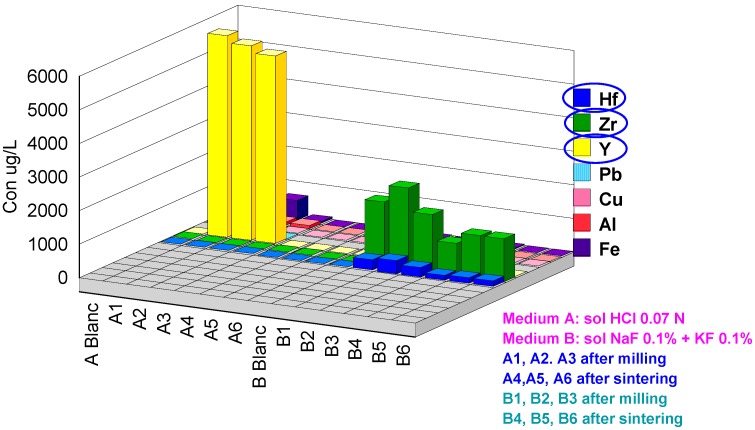
Cations released in the thousandth µg/L scale.

Figure 5 presents the concentrations of released cations in the thousandth µg/L scale. The zirconia in the fluorinated medium is released from the two physical states of matter in quantities of 1000 to 2400 µg/L. Regardless of the after milling or after sintering state, the quantities of zirconia are of the same order. It is the fluorocomplex zirconia formed in the presence of F-ions which is soluble in the extraction medium [33,34].

On the other hand, in the acid medium (HCl, concentration 0.07 M) the after milling samples release small quantities: 14, 4.2 and 10 µg/L. For the after-sintering samples the quantities found are at the limit of ICP-OES or ICP-MS detection.

Another cation found in the same magnitude order is Y. The concentrations measured in the acid medium for the after milling samples are A1: 6000 µg/L, A2: 5800 µg/L and A3: 5600 µg/L. The solubility of yttrium chloride (III) is 751 g/L (20 °C) in water [35], 601 g/L(15 °C) in ethanol [36] and 606 g/L (15 °C) in pyridine [37].

**Figure 6:**
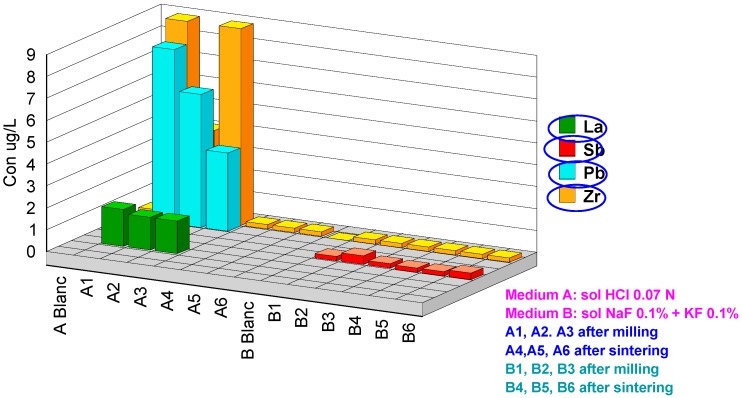
Cations released in the µg/L scale.

For the after-sintering samples, the values are 6.2, 4.4 and 3.2 µg/L (Figure 6). It seems that the sintering process greatly decreases the amounts of yttrium released, probably caused by a modification in the morphological structure of the ceramic.

In case of fluorapatite glass-ceramics the heat treatment temperature strongly influences microstructure and crystallinity. Heat treatment temperatures should remain below 1100 °C, together with slow heating rates, to prevent crystal dissolution, and preserve the microstructure [38]. On the other hand, at low temperature, the fluorapatite glass-ceramics are traditionally difficult to sinter. Increasing the Ca/Al ratio promotes low temperature sintering of fluorapatite glass-ceramics [39].

In the fluorinated medium, the quantities of yttrium are at the detection limit of the ICP. Yttrium fluoride (III) is insoluble in an aqueous medium <0.03 mg/L [24].

In the µg/L scale Pb, Sb, La and Zr have been detected (Figure 6). The La and Pb cations are released by the after milling samples in the acid medium. In the fluoride medium, the concentrations of Pb and La are at the detection limit. In the fluoride medium the released quantities of Zr are of the order of a thousandth (Figure 5).

**Figure 7:**
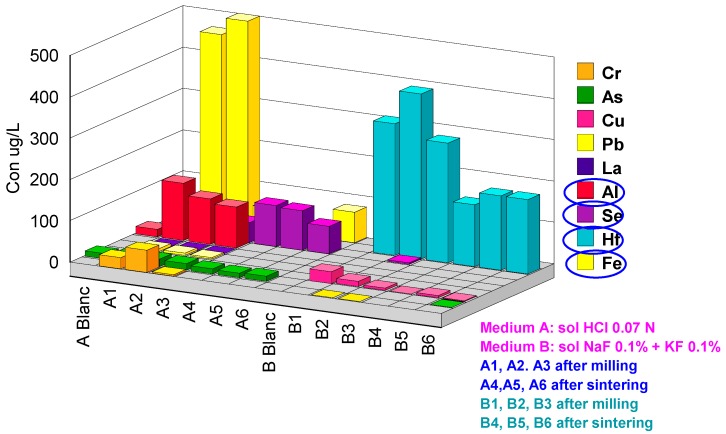
Cations released in the hundredth µg/L scale.

In the hundredth µg/L scale (Figure 7), in the acid medium, values for Al are: A1: 140 µg/L, A2: 110 µg/L, A3: 99 µg/Land for Fe:A1: 460 µg/L, A2: 585 µg/L, A3: 56 µg/L. The solubility of Al and Fe chlorides in the HCl solution is important as it explains the quantities found. On the other hand, after the sintering process, the quantities are at the detection limit. In the fluoride environment we find Hf. All samples B1–B6 release quantities which vary from 150 µg/L to 500 µg/L.

**Figure 8:**
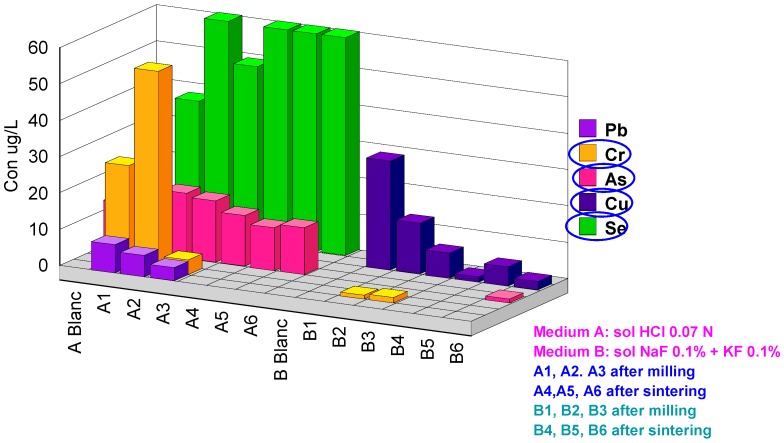
Cations released in the tenth of µg/L scale.

In the tenth µg/L scale (Figure 8) Cr, Se, As and Cu are to be found. These metals are probably found as impurities in the components of the ceramic. As and Se, which are toxic elements, are present in all samples (after milling and after sintering) in the 0.07M HCl acid solution (Table 3). 

Se has proven to be toxic when ingested, according to existent studies toxic values revealed in blood are: 479 µg/L of serum, 8 weeks after ingestion, and 300 µg/L of serum, 9 weeks after ingestion [40].

Severe symptoms have also been frequently observed in individuals with longer term lower dose exposures via drinking water (exposure levels between 0.050 and 0.393 mg/L). These effects are usually not detectable at medium term and generally decline within a short time after exposure ceases [41]. 

It also should be considered that, in case of dental applications, the exposed people are in prolonged contact with the bioceramic devices.

It is easily noticeable that, according to the results obtained, the ceramic evaluated is not really inert.

### 3.2. Ceramics Intended to Come inContact with Skin

#### 3.2.1. Cermet Ceramic

A cermet is a composite material composed of ceramic (cer) and metal (met) materials. The most used metals are Ni, Mo, Co, Cr and Fe.

The investigations have been carried out on six cermet rings, samples C1–C6. SamplesC1, C2 and C3 to be analyzed have been immersed in artificial sweat according to EN 1811 [29]. Samples C4, C5 and C6 have been immersed in an acid HCl 0.07M solution [26,27]. All samples have been immersed for 7 days at 30 °C with a volume/surface rate of 10 mL for 1.7 cm^2^. Following the extraction test, samples C4 and C5 have been dissolved in an acid mixture. The dissolved mass of samples C4 and C5 is 0.624104 g and 0.595528g respectively.

The ICP analysis of the composition of the cermet rings, presented in Table 4, reveals:

- major elements: Ni, Cr and a lower concentration of Co and Fe (minor elements).
- traces: some of which exceed 5 ppm (Hg, Te and Cu). The Hg concentration reaches 100 ppm and Cu 13 ppm.

The results show that this cermet is doped with Ni and Cr. Analysis of the composition reveals the presence of toxic elements. The Hg content is 100 ppm, that of Te 9 ppm and that of As 3 ppm. Other toxic elements present in quantities less than 1 ppm are Se, Fe, Cd and Sb.

Table 5 presents the extraction results in the artificial sweat medium. In artificial sweat, the release of Ni amounts to approximately 0.3 µg/cm^2^; traces of Ba, Cr and Pb are also present.

Table 6 presents the extraction results in the HCl 0.07M solution. In hydrochloric acid, the release of Ni amounts to approximately 2.5 µg/cm^2^ and of Cr to 0.1 µg/cm^2^.These values how that, in contact with the skin, there is a risk of inducing a contact allergy.

The screening tests are negative (Table 7), this is due to the sensitivity of the reagents. For dimethylglyoxime against Ni, for example, the concentration limit is 2 mg/L (corresponding to a release of 22 µg/cm^2^·week). The extractions results (Table 5; Table 6) suggest that the reagents sensitivity is insufficient to reveal the release with a screening test.

Comments: The cermet rings tested release Ni. However, this release is less than 0.5 µg/cm^2^·week. For the moment, there is no information regarding the possible consequences of the presence of certain elements found in objects which come in contact with the skin, but they are inherently toxic.

#### 3.2.2. Zirconia Ceramics for Watch making Applications: White Ceramic Watch Link and Black Ceramic Watch Band

**Figure 9:**
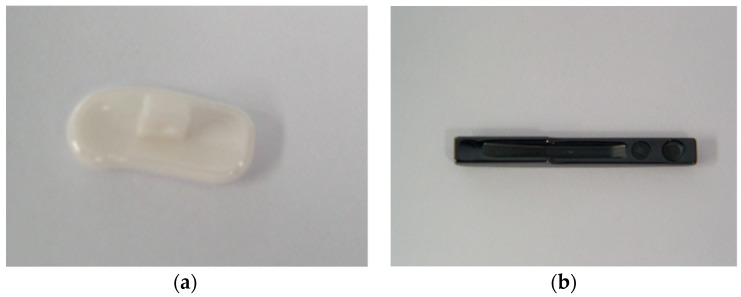
(**a**)White ceramic watch link D1. (**b**)Black ceramic watch bandD2.

Investigations have been focused on two types of zirconia ceramics: white D1 and blackD2, used as components in watches manufacturing. (Figure 9a,b)

Two samples of each type of zirconia ceramic (Table 8) have been dissolved under ultrasound in an acid mixture for 16h.After dilution, the filtrates have been analyzed by ICP-MS. Scanning electron microscope (SEM) semi-quantitative analysis of microfibers from the filter paper was carried out.

The results for the solutions set analyzed are presented in Table 9.

**Figure 10:**
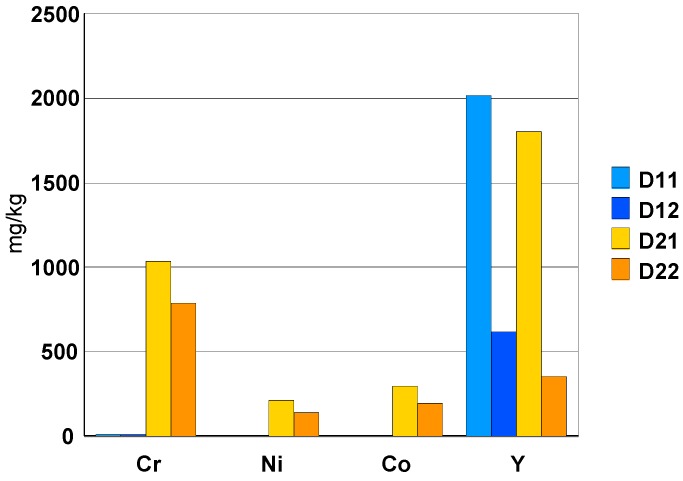
Concentrations of Cr, Ni, Co and Y [mg/kg] found in solution.

The black ceramic watch band D2 contains Cr, Ni and Co in concentrations between 200 mg/kg and 1000 mg/kg (Figure 10). The concentration of Y is very variable because this element precipitates in the form of fluoride and the solubility parameters vary from one dissolution to another (ratio volume/mass of sample, effective ultrasonic power, etc.).

**Figure 11:**
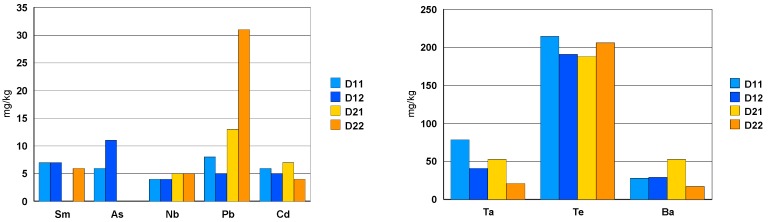
Concentration of the trace elements [mg/kg] found in solution.

With the exception of Pb (Figure 11), the concentration of trace elements is roughly the same for the two types of ceramic. The Te concentration is around 200 mg/kg (Figure 10).

**Figure 12:**
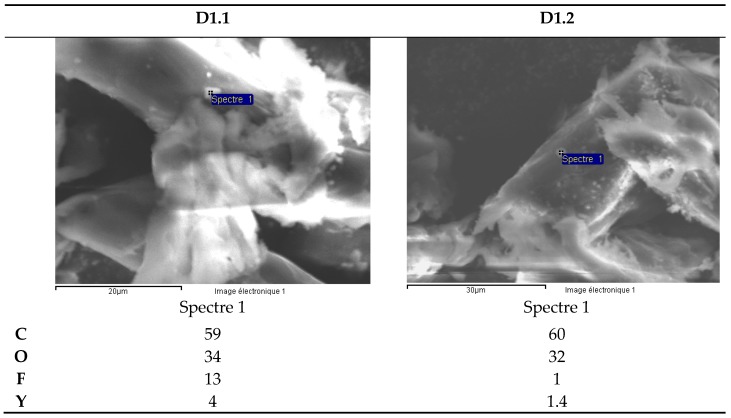
White ceramic watch link (D1.1 and D1.2). Scanning electron microscope (SEM) analysis of microfibers from filter paper.

SEM analysis of microfibers from the filter paper for the white ceramic watch link reveals the presence of yttrium fluoride precipitates (Figure 12). The presence of C and O is likely due to the cellulose in the filter microfibers.

**Figure 13:**
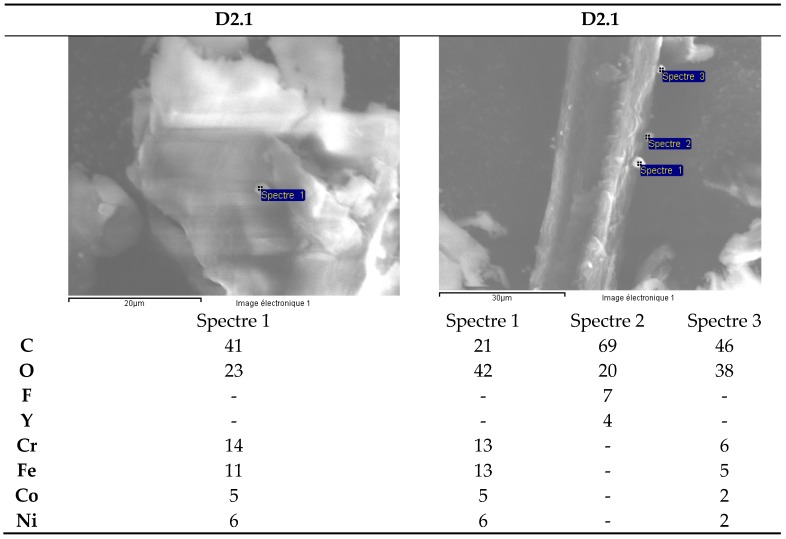
Black ceramic watch band (D2.1). SEM analysis of microfibers from filter paper.

**Figure 14:**
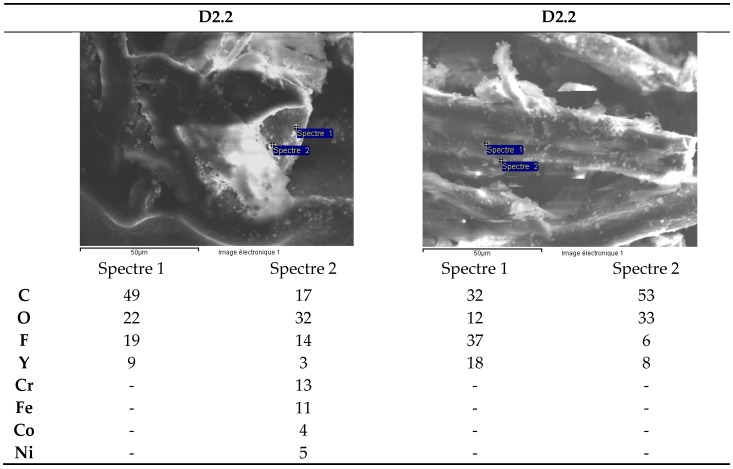
Black ceramic watch band (D2.2). SEM analysis of microfibers from filter paper.

SEM analysis of the precipitates from the dissolution of the black ceramic watch band D2 shows, apart from yttrium fluorides, the presence of Fe, Cr, Ni, Co particles (Figure 13; Figure 14).

Screening tests of elements Ni, Co, Cu and Fe were carried out. The detection limits for Merckoquant^®^ type strips are shown in Table 10. 

**Figure 15:**
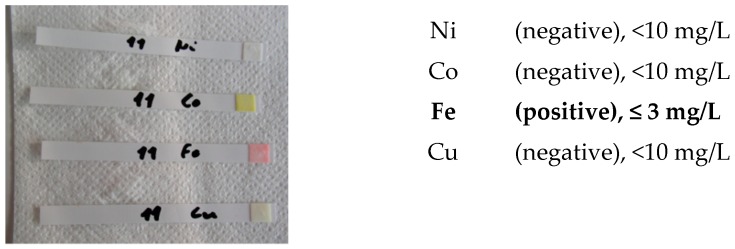
Screening test. White ceramic watch link.

**Figure 16:**
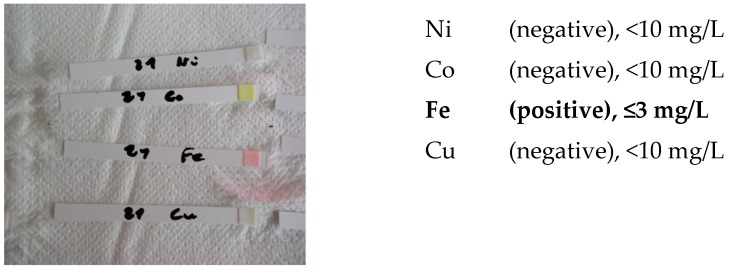
Screening test. Black ceramic watch band.

Both white ceramic watch link and black ceramic watch band are Fe positive (Figure 15 and Figure 16).

Comments

The trace elements in the composition of the two ceramics can be classified as follows:

- elements: Hf (1.5%), Y (0.2% + precipitation as fluoride), Ta (about 50 mg/kg) and Nb (5 mg/kg).
- toxic elements such as Te (0.02% = 200 mg/kg) and Ba (30 mg/kg) as well asAs, Pb and Cd with a concentration between 5 and 30 mg/kg.
- among the rare earths, only Sm was measured in a concentration of about 7 mg/kg.
- elements: Cr (0.1%), Ni (0.02%) and Co (0.02%) are present in the composition of the black ceramic watch band.

Upon dissolution in the HF/HNO_3_ mixture, yttrium fluoride precipitates. For the black ceramic watch band, Fe, Cr, Ni, Co particles are identified in addition to yttrium fluoride. All these particles have the same chemical composition: Fe (35%), Cr (35%), Co (15%) and Ni (15%).

Generally, these zirconia ceramics contain a lot of trace elements, some of which are toxic or very toxic (Sm); therefore, they are labeled as technical zirconia ceramics, not intended for the medical field. The particles of Fe, Cr, Co and Ni, found in the black ceramic watch band are probably due to the pigmentation of the ceramic. The insoluble analysis technique did not make it possible to determine the presence of C in the black ceramic watch band D2, as C cannot be detected by SEM analysis. The screening test on the black ceramic did not reveal the release of Co and Ni because the detection limit for these elements is 10 mg/L (Table 9). Considering the composition of these ceramics, release into artificial sweat of toxic elements should be further considered.

#### 3.2.3. Black Zirconia Ceramic Bracelet Component

**Figure 17:**
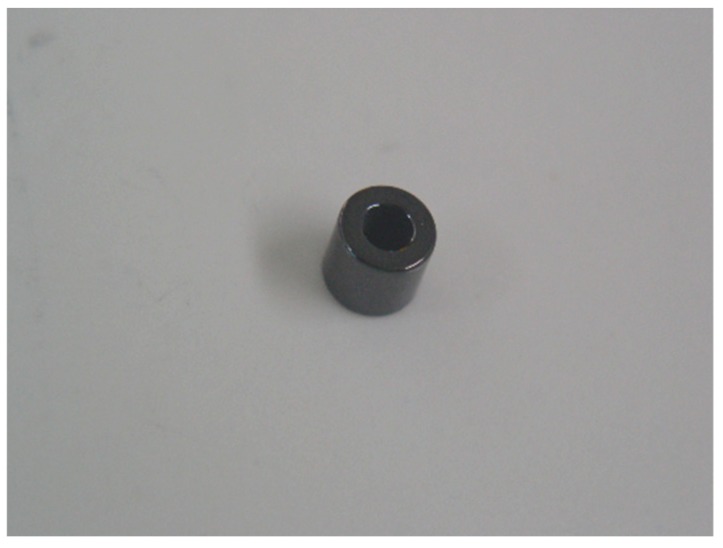
Black ceramic bracelet component E.

The investigations were carried out on two samples of black zirconia ceramic bracelet components E (Figure 17).

Two samples the black zirconia ceramic bracelet components, E1 and E2 (Table 11) have been dissolved under ultrasound in an acid medium for 16h and the composition was analyzed by ICP-MS.

The results of trace analysis in solution are shown in Table 12.

**Figure 18:**
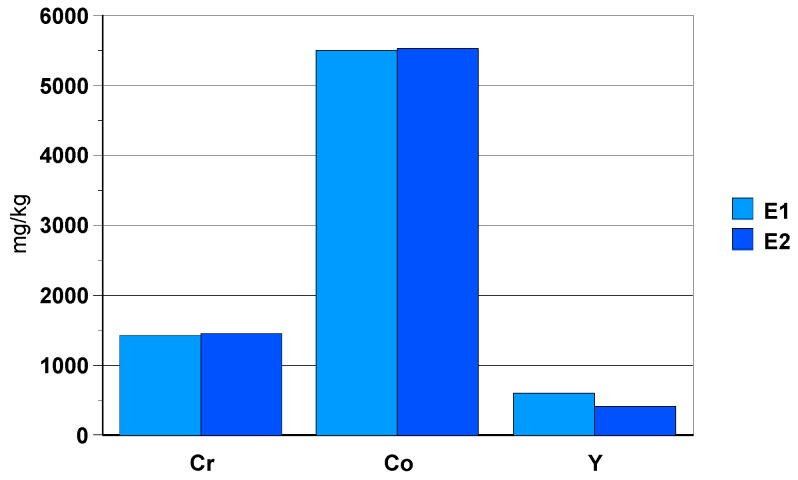
Concentration of elements Cr, Ni, Co and Y [mg/kg] found in solution.

The black zirconia ceramic bracelet components present Co in a concentration of 5000 mg/kg, Cr 1400 mg/kg and Y 500 mg/kg (Figure 18). The concentration of Y is variable because this element precipitates in the form of fluoride and the solubility parameters vary from one dissolution to another (ratio volume mass of sample, effective ultrasonic power, etc.).

**Figure 19:**
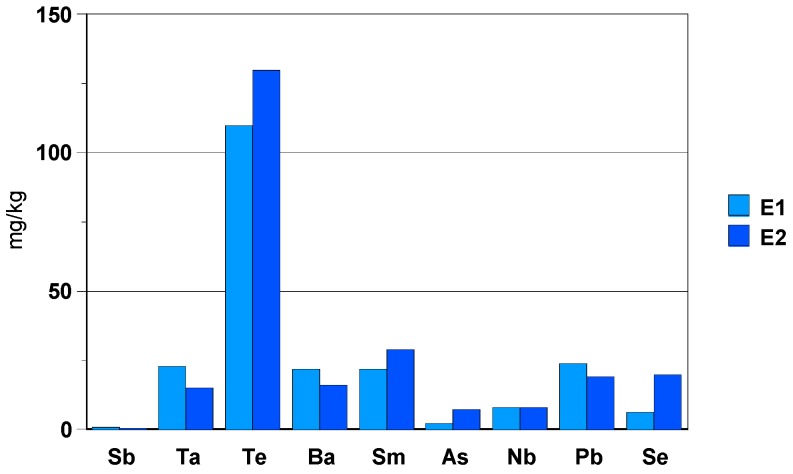
Concentration of trace elements [mg/kg] found in solution.

The Te concentration is around 120 mg/kg; for the other trace elements, the concentration is less than 30 mg/kg. The concentration of Sb, As and Nb is very close to the detection limit of ICP-MS (Figure 19).

**Figure 20:**
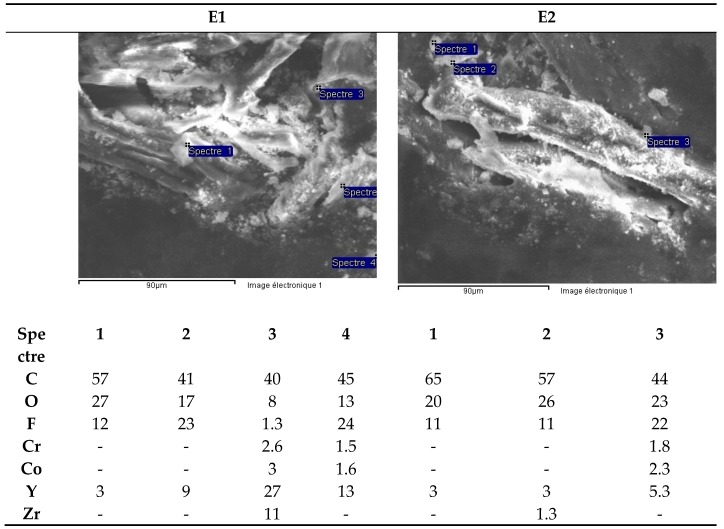
Black zirconia ceramic bracelet components (E1 and E2). SEM analysis of microfibers from filter paper.

The semi-quantitative SEM analysis of the microfibers of the filter paper essentially reveals the presence of yttrium fluoride precipitates (Figure 20) and traces of Cr and Co. The presence of C and O is likely due to the cellulose in the filter microfibers.

**Figure 21:**
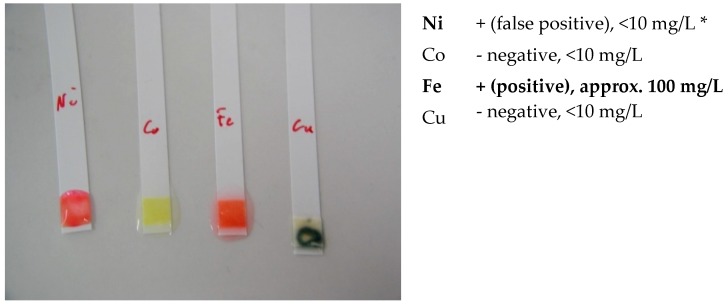
Screening tests. Black zirconia ceramic bracelet components E1 and E2. * Fe, in a concentration above 10 mg/l interferes with Ni.

The black zirconia ceramic bracelet components are positive for Fe (Figure 21). Co has not been detected during the screening test (<10 mg/L) (Figure 19), despite a concentration of 0.55% in the alloy (Table 12).

Comments

The trace elements in the composition of the two black ceramic bracelet component samples can be classified as follows:

- elements: Hf (3wt.%), Y (0.05wt.%+fluoride precipitation), Ta (aprox. 10 mg/kg) and Nb (4 mg/kg).During dissolution in the HF/HNO_3_ mixture, yttrium fluoride precipitates.
- toxic elements such as Te (50 mg/kg) and Ba (10 mg/kg), as well asSm and Pb with a concentration of approx. 10 mg/kg.
- elements: Cr (0.1%) and Co (0.5%) are present in the composition of the black zirconia ceramic bracelet component.

The screening test did not reveal the release of Co because the detection limit is 10 mg/L.

This type of black zirconia ceramic contains a lot of trace elements, some of which are toxic or very toxic (Sm); therefore, this technical zirconia ceramic is not intended for the medical field.

#### 3.2.4. Zirconia Ceramic Rings, Jewelry Components, Two Black and One White (Figure 22)

ICP-OES and ICP-MS trace analysis of three different zirconia ceramic rings F1–F3, for jewelry applications, dissolved in an acid mixture, were performed.

**Figure 22:**
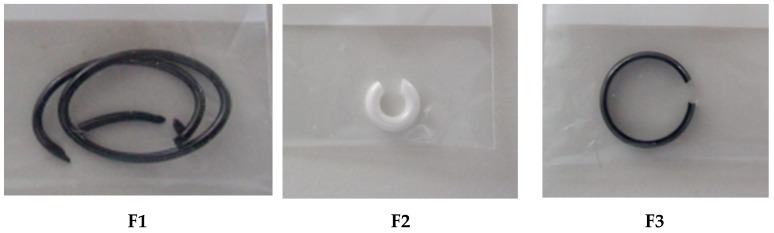
Zirconia ceramic rings: samples F1, F2 and F3.

A sample of each zirconia ceramic ring (Figure 22) has been dissolved and the solution analyzed by ICP-OES and ICP-MS. Table 13 presents the dissolved masses and the dissolution times.

The chemical compositions of the three zirconia ceramic rings are shown in Table 14.

In a first approach we notice that the studied ceramics contain many trace elements. Is it reasonable to use articles with such composition in prolonged contact with the skin?

**Figure 23:**
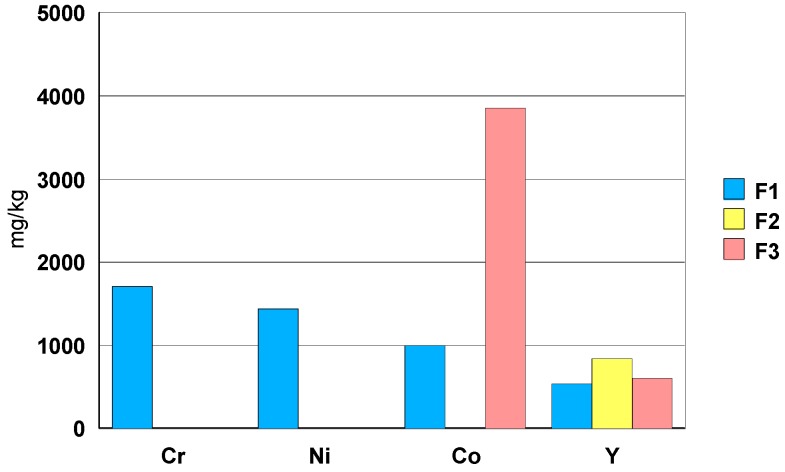
Concentration of elements Cr, Ni, Co and Y [mg/kg] found in solution.

The ceramic F1 presents, in its composition, Cr, Ni and Co in concentrations between 1000 mg/kg (=0.1wt.%) and 1700 mg/kg (Figure 23). The ceramic F3shows a Co concentration close to 4000 mg/kg (=0.4wt.%). Part of the Y is precipitated in the form of fluoride, it remains in solution between 400 and 2000 μg/L.

**Figure 24:**
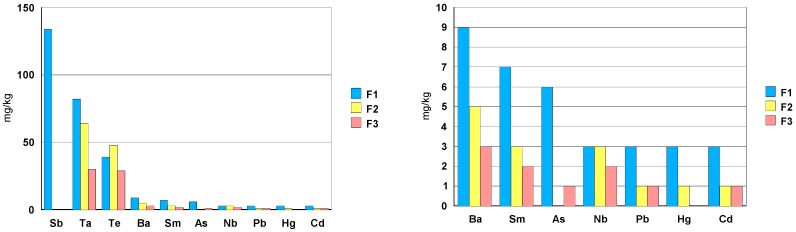
Concentration of trace elements [mg/kg] found in solution.

Sample F1 also contains Sb in a quantity of 130 mg/kg. As for other trace elements, the concentrations are systematically higher for ceramic F1 (Figure 24).

**Figure 25:**
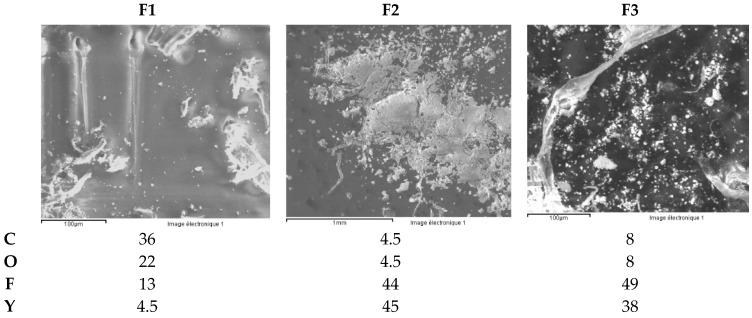
SEM analysis of the precipitates [%m/m].

The precipitates are mainly composed of yttrium fluoride (Figure 25). For sample F1, the SEM analysis reveals C, which probably comes from the black coloring of the ceramic (Figure 24).

Comments

**Figure 26:**
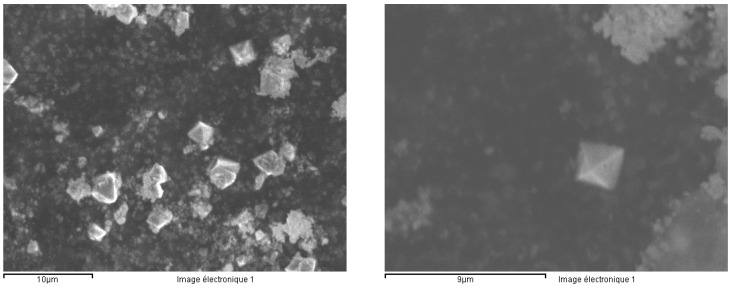
Yttrium fluoride crystals.

Yttrium fluorides are insoluble in a hydrofluoric acid medium, they form octahedral crystals (Figure 26).

In general, the composition of the three zirconia ceramic rings, jewelry components, can be described as follows:

- elements: Hf, Y, Nb and Ta.
- toxic trace elements: Ba, As, Pb, Te, Cd and Hg in a concentration between 1mg/kg and 10 mg/kg.
- ceramic F1 shows a Sb concentration of about 100 mg/kg.
- among the rare earths, only Sm has been detected with a concentration of about 7 mg/kg.
- in case of ceramic F1, the elements Cr, Ni and Co have a concentration order of 0.1wt.% (1000 mg/kg). Ceramic F3shows a Co concentration close to 0.4 wt.% (4000 mg/kg).

In a hydrofluoric acid medium, yttrium precipitates in the form of fluoride. For ceramic F1, the precipitate also contains C which probably comes from the black coloring of the ceramic.

Generally, these zirconia ceramic rings, jewelry components, contain a lot of trace elements; therefore, being technical zirconia ceramics which are not intended for the medical field. From the composition point of view, ceramic F2 is the most suitable for usage in contact with the skin. Indeed, it does not contain either Ni or Cr (found in F1) or Co (found in F1 and F3). However, an extraction test in artificial sweat may give information on the release in contact with the skin.

## 4. Conclusions

Generally speaking, the ceramic materials “are inert in the corrosion process”, compared with most of the other materials. No large-scale results research works on the corrosion behavior of zirconia ceramic have been available so far.

Concerning this subject, two aspects have to be considered:

The stability of zirconia over time, the tetragonal stable phase being transformed in an unstable monoclinic phase.

The multitude of manufacturing processes, with diverse added additives (as sintering additives), the processes of mixture for oxides, impurities, gaseous phases in the joints of grains, and the porosities may strongly influence the corrosion and the chemical degradation using various additives (such as sintering additives), oxides mixing, impurities, gas phases at grain boundaries, and porosities which strongly influence the corrosion behavior and chemical degradation.

The ceramics studied have been proven as not “inert”, as all of them release cations traces, with potential short- or long-term toxic consequences.

The question concerning their toxicological behavior still remains: What are the long term consequences in humans?

## Figures and Tables

**Table 1:** Types of ceramics for medical applications.

Class	References	Form and Composition
Alumina-based ceramic	ISO 6574	Dense sintered crystalline alumina A1203
		99.9% porous alumina
		97% pure porous alumina
		Zirconia reinforced alumina
		Calcium-phosphate alumina
		Single crystal alumina
Silicate-based ceramic		Porous vitroceramic
		Dense vitroceramic
Calcium-phosphate-based ceramic		Dense sintered hydroxy-apatite
		Porous sintered hydroxy-apatite
		Dense sintered beta tricalcium phosphate
		Porous sintered beta tricalcium phosphate
		Other types
Calcium carbonate-based ceramic		Porous sintered calcium carbonate
		Natural porous calcium carbonate
Zirconia-based ceramic		Alumina stabilized zirconia
		Yttria partially stabilized zirconia
		Magnezium stabilized zirconia
		Ceria partially stabilized zirconia
Felds pat-based ceramic	ISO 6872 ISO 9693	Reinforced with leucit
		Reinforced with alumina
		Reinforced with mica

**Table 2:** Properties of zirconiadental ceramics.

Property	Y-TZP [12,13]	ISO 13356 [14]	Zenostar [15]
Chemical composition			
ZrO_2_+HfO_2_+Y_2_O_3_	>99.0	>99.0	>99.0
Y2O_3_	4.5–5.4	4.5–5.4	>4.5
Al_2_O_3_	<0.5	<0.5	<0.5
Other total oxides	<0.5	<0.5	<1.5
Physical properties			
Bulk density (g/cm^3^)	6.05	>6.00	>6.00
Grainsize (μm)	0.2	<0.6	<0.6
Monoclinic phase (%)	1	-	<5
Mechanical properties			
Flexural strength-4 point bend (MPa)	1666	>800	1200±200
Elastic modulus (GPa)	201	-	210
Vickers hardness (HV)	1270	-	>1300
Fracture toughness (Kgf/mm^2/3^)	16.8	-	-
Compressive strength (MPa)	4900	-	-
Impact strength (MPa)	137	-	-

**Table 3:** The extraction results [µg/L].

Code	Hf	Cr	Y	As	Pb	Al	Fe	Cu	Se	Zr	Sb	La
ABlanc	<0.2	<1	<0.2	13	<0.2	19	<20	<0.2	<1	<0.2	<0.2	<0.2
A1	1.9	28	6000	16	8.0	140	460	<0.2	37	14	<0.2	1.7
A2	0.2	55	5800	18	6.1	110	585	<0.2	63	4.2	<0.2	1.5
A3	<0.2	4.2	5600	17	3.6	99	56	<0.2	49	10	<0.2	1.5
A4	<0.2	<1	6.2	14	<0.2	<10	<20	<0.2	100	<0.2	<0.2	<0.2
A5	<0.2	<1	4.4	12	<0.2	<10	<20	<0.2	97	<0.2	<0.2	<0.2
A6	<0.2	<1	3.2	13	<0.2	<10	75	<0.2	67	<0.2	<0.2	<0.2
BBlanc	<0.2	<1	<0.2	<1	<0.2	<10	<20	<0.2	<1	3.1	<0.2	<0.2
B1	320	<1	<0.2	<1	<0.2	<10	<20	30	<1	1900	0.2	<0.2
B2	400	1.2	<0.2	<1	<0.2	<10	<20	14	4.2	2400	0.4	<0.2
B3	290	1.5	<0.2	<1	<0.2	<10	<20	<0.2	<1	1700	0.2	<0.2
B4	150	<1	<0.2	<1	<0.2	<10	24	0.5	<1	960	0.2	<0.2
B5	180	<1	<0.2	<1	<0.2	<10	<20	5.4	<1	1300	0.2	<0.2
B6	180	<1	<0.2	1.1	<0.2	<10	<20	2.5	<1	1300	0.3	<0.2

**Table 4:** Inductively coupled plasma(ICP) analysis of the composition.

Element	Method	Blanc	C4			C5		
		µg/L	µg/L	% (m/m)	ppm	µg/L	% (m/m)	ppm
Ni	ICP-OES	<20	646,000	10.4	103,508	634,000	10.6	10,6460
Cr	ICP-OES	<5	142,000	2.27	22,753	135,000	2.27	22,669
Co	ICP-OES	<10	4370	0.07	700	4150	0.07	697
Fe	ICP-OES	<20	1900	0.03	304	1870	0.03	314
Hg	ICP-MS	<0.5	570	-	91	610	-	102
Cu	ICP-MS	<0.2	81	-	13	78	-	13
Te	ICP-MS	<1	40	-	6.4	52	-	8.7
As	ICP-MS	<1	22.0	-	3.5	15.0	-	2.6
Ba	ICP-MS	<0.2	10.0	-	1.6	8.8	-	1.5
Se	ICP-MS	<1	4.1	-	0.7	4.5	-	0.7
Pb	ICP-MS	<0.2	3.0	-	0.5	3.0	-	0.5
Cd	ICP-MS	<0.2	1.9	-	0.3	1.6	-	0.3
Sb	ICP-MS	<0.2	3.5	-	0.6	1.7	-	0.3

**Table 5:** ICP analysis results in the artificial sweat extraction solution. DL: Detection limit.

Element	Method	Blanc	C1		C2		C3	
		µg/L	µg/L	µg/cm^2^	µg/L	µg/cm^2^	µg/L	µg/cm^2^
Fe	ICP-OES	<20	<20	DL	<20	DL	<20	DL
Co	ICP-OES	<10	<10	DL	<10	DL	<10	DL
Cu	ICP-OES	<20	<20	DL	<20	DL	<20	DL
Ni	ICP-OES	<20	49	0.29	48	0.28	57	0.34
As	ICP-MS	<1	<1	DL	<1	DL	<1	DL
Ba	ICP-MS	4.4	6.3	0.037	5.4	0.032	5.4	0.032
Cd	ICP-MS	<0.2	<0.2	DL	<0.2	DL	<0.2	DL
Cr	ICP-MS	<1	4.1	0.024	4.0	0.024	4.4	0.026
Hg	ICP-MS	<0.5	<0.5	DL	<0.5	DL	<0.5	DL
Pb	ICP-MS	<0.5	0.9	0.005	0.9	0.005	0.9	0.005
Sb	ICP-MS	<0.2	<0.2	DL	<0.2	DL	<0.2	DL
Se	ICP-MS	<1	<1	DL	<1	DL	<1	DL
Te	ICP-MS	<1	<1	DL	<1	DL	<1	DL

**Table 6:** ICP analysis of extraction results in the HCl 0.07 M solution. DL: Detection limit.

Element	Method	Blanc	C4		C5		C6	
		µg/L	µg/L	µg/cm^2^	µg/L	µg/cm^2^	µg/L	µg/cm^2^
Fe	ICP-OES	<20	<20	DL	<20	DL	<20	DL
Co	ICP-OES	<10	<10	DL	<10	DL	<10	DL
Cu	ICP-OES	<20	<20	DL	<20	DL	<20	DL
Ni	ICP-OES	<20	433	2.6	407	2.4	421	2.5
As	ICP-MS	<1	<1	DL	<1	DL	<1	DL
Ba	ICP-MS	<0.2	1.3	0.008	1.2	0.007	6.2	0.036
Cd	ICP-MS	<0.2	<0.2	DL	<0.2	DL	<0.2	DL
Cr	ICP-MS	<1	15.0	0.09	17.0	0.10	14.0	0.08
Hg	ICP-MS	<0.5	<0.5	DL	<0.5	DL	<0.5	DL
Pb	ICP-MS	<0.2	0.4	0.002	0.6	0.004	0.5	0.003
Sb	ICP-MS	<0.2	<0.2	DL	<0.2	DL	<0.2	DL
Se	ICP-MS	<1	<1	DL	<1	DL	<1	DL
Te	ICP-MS	<1	<1	DL	<1	DL	<1	DL

**Table 7:** Results of screening tests. Negative = not detected.

Element	Lower Measurement Limit [mg/L]	4h	18h
Ni	2	Negative	Negative
Co	2	Negative	Negative
Cu	2	Negative	Negative
Fe	3	Negative	Negative

**Table 8:** Mass of test samples.

Description	Code	Mass [g]
White ceramic watch link	D1.1	0.03715
	D1.2	0.03398
Black ceramic watch band	D2.1	0.01888
	D2.2	0.03888

**Table 9:** Trace analysis in the solutions; Analysis results and conversion in units [mg/kg] and [%]. 0.01% = 100 mg/kg. DL: Detection limit.

White Ceramic Watch Link D1								Black Ceramic Watch Band D2							
D1.1	µg/L	mg/kg	%	D1.2	µg/L	mg/kg	%	D2.1	µg/L	mg/kg	%	D2.2	µg/L	mg/kg	%
Hf	12,000	16,151	1.62	Hf	9900	14,567	1.46	Hf	5700	15,095	1.51	Hf	12,000	15,432	1.54
Cr	7.3	10	-	Cr	5.4	8	-	Cr	390	1033	0.10	Cr	790	1016	0.10
Ni	<20	DL	DL	Ni	<20	DL	DL	Ni	80	212	0.02	Ni	137	176	0.02
Co	<10	DL	DL	Co	<10	DL	DL	Co	111	294	0.03	Co	194	249	0.02
Y	1500	2019	0.20	Y	420	618	0.06	Y	680	1801	0.18	Y	350	450	0.05
Sb	<0.4	DL	DL	Sb	<0.4	DL	DL	Sb	<0.4	DL	DL	Sb	<0.4	DL	DL
Ta	59	79	0.008	Ta	28	41	0.004	Ta	20	53	0.005	Ta	16	21	0.002
Te	160	215	0.02	Te	130	191	0.02	Te	71	188	0.02	Te	160	206	0.02
Ba	21	28	-	Ba	20	29	-	Ba	20	53	0.01	Ba	13	17	-
Sm	5.0	7	-	Sm	4.6	7	-	Sm	<2	DL	DL	Sm	4.4	6	-
As	4.5	6	-	As	7.5	11	-	As	<2	DL	DL	As	<2	DL	DL
Nb	3.0	4	-	Nb	2.4	4	-	Nb	1.8	5	-	Nb	3.6	5	-
Pb	5.7	8	-	Pb	3.5	5	-	Pb	4.9	13	-	Pb	24	31	-
Hg	<1	DL	DL	Hg	<1	DL	DL	Hg	<1	DL	DL	Hg	<1	DL	DL
Cd	4.5	6	-	Cd	3.1	5	-	Cd	2.7	7	-	Cd	2.9	4	-
Cu	<20	DL	DL	Cu	<20	DL	DL	Cu	<20	DL	DL	Cu	<20	DL	DL
Se	<2	DL	DL	Se	<2	DL	DL	Se	<2	DL	DL	Se	<2	DL	DL

**Table 10:** Reagents and detection limit (Merck^TM^).

Element	Reagent	Detection Limit (Semi-Quantitative)
Ni	Diméthylglyoxime	10 mg/L
Co	Thiocyanate	10 mg/L
Fe	2,2′-bipyridine	3 mg/L
Cu	2,2′-biquinoléine	10 mg/L

**Table 11:** Mass of test samples.

Code	Mass [g]
E1	0.10807
E2	0.12027

**Table 12:** Inductively coupled plasma-mass spectrometry (ICP-MS) analysis results and conversion to units [mg/kg] and [%]. 0.01% = 100 mg/kg. DL: detection limit.

Black Zirconia Ceramic Bracelet Components							
E1	μg/L	mg/kg	%	E2	μg/L	mg/kg	%
Hf	64,000	29,610	3.0	Hf	74,000	30,764	3.08
Cr	3100	1434	0.14	Cr	3500	1455	0.15
Ni	<80	DL	DL	Ni	<80	DL	DL
Co	11,900	5506	0.55	Co	13,300	5529	0.55
Fe	8810	4053	0.41	Fe	9925	4163	0.42
Y	1300	601	0.06	Y	990	412	0.04
Sb	0.9	0.4	-	Sb	0.5	0.2	-
Ta	23	11	-	Ta	15	6	-
Te	110	51	-	Te	130	54	-
Ba	22	10	-	Ba	16	7	-
Sm	22	10	-	Sm	29	12	-
As	2.3	1	-	As	7.3	3	-
Nb	7.8	4	-	Nb	8.0	3	-
Pb	24	11	-	Pb	19	8	-
Hg	≤1	DL	DL	Hg	≤1	DL	DL
Cd	≤8	DL	DL	Cd	≤8	DL	DL
Cu	≤80	DL	DL	Cu	≤80	DL	DL
Se	6.4	3	-	Se	19.8	8	-

**Table 13:** Test samples. Mass and dissolution time.

Sample	Mass [mg]	Dissolution Time [h]	Color of Precipitates
F1	8180	16 (ultrasound)	blackish
F2	24,923	40 (ultrasound)	white
F3	16,134	160 (ultrasound)	white

**Table 14:** ICP-OES and ICP-MS analysis results and conversion to units [mg/kg] and [%].

F1	μg/L	mg/kg	%	F2	μg/L	mg/kg	%	F3	μg/L	mg/kg	%
Hf	12,000	14,670	1.47	Hf	46,000	18,457	1.85	Hf	27,000	10,833	1.08
Cr	1400	1711	0.17	Cr	3.9	2	-	Cr	3.5	1	-
Ni	1180	1443	0.14	Ni	<20	-	-	Ni	<20	-	-
Co	820	1002	0.10	Co	<10	-	-	Co	9610	3856	0.39
Y	440	538	0.05	Y	2100	843	0.08	Y	1500	602	0.06
Sb	110	134	0.01	Sb	<0.4	-		Sb	<0.4	-	-
Ta	67	82	0.01	Ta	160	64	0.01	Ta	76	30	-
Te	32.0	39	-	Te	120	48	-	Te	73	29	-
Ba	7.7	9	-	Ba	12	5	-	Ba	8.0	3	-
Sm	6.0	7	-	Sm	8.6	3	-	Sm	6.2	2	-
As	4.5	6	-	As	<2	-	-	As	2.4	1	-
Nb	2.6	3	-	Nb	8.2	3	-	Nb	6.0	2	-
Pb	2.4	3	-	Pb	2.1	1	-	Pb	1.3	1	-
Hg	2.4	3	-	Hg	1.8	1	-	Hg	<1	-	-
Cd	2.3	3	-	Cd	3.5	1	-	Cd	2	1	-
Cu	<20	-	-	Cu	<20	-	-	Cu	<20	-	-
Se	<2	-	-	Se	<2	-	-	Se	<2	-	-
